# Measles Outbreak with Unique Virus Genotyping, Ontario, Canada, 2015

**DOI:** 10.3201/eid2307.161145

**Published:** 2017-07

**Authors:** Shari Thomas, Joanne Hiebert, Jonathan B. Gubbay, Effie Gournis, Jennifer Sharron, Alberto Severini, Manisa Jiaravuthisan, Amanda Shane, Valerie Jaeger, Natasha S. Crowcroft, Jill Fediurek, Beate Sander, Tony Mazzulli, Helene Schulz, Shelley L. Deeks

**Affiliations:** Public Health Ontario, Toronto, Ontario, Canada (S. Thomas, J.B. Gubbay, N.S. Crowcroft, J. Fediurek, B. Sander, T. Mazzulli, S.L. Deeks);; Public Health Agency of Canada, Winnipeg, Manitoba, Canada (J. Hiebert, A. Severini);; University of Toronto, Toronto (E. Gournis, N.S. Crowcroft, B. Sander, T. Mazzulli, S.L. Deeks);; Niagara Region Public Health, Niagara Falls, Ontario, Canada (J. Sharron, V. Jaeger);; Toronto Public Health, Toronto (M. Jiaravuthisan);; Public Health Agency of Canada, Ottawa, Ontario, Canada (A. Shane);; University of Manitoba, Winnipeg (A. Severini, H. Schulz)

**Keywords:** measles, outbreak, public health, Canada, Ontario, genotyping, extended sequencing, viruses

## Abstract

The province of Ontario continues to experience measles virus transmissions despite the elimination of measles in Canada. We describe an unusual outbreak of measles in Ontario, Canada, in early 2015 that involved cases with a unique strain of virus and no known association among primary case-patients. A total of 18 cases of measles were reported from 4 public health units during the outbreak period (January 25–March 23, 2015); none of these cases occurred in persons who had recently traveled. Despite enhancements to case-patient interview methods and epidemiologic analyses, a source patient was not identified. However, the molecular epidemiologic analysis, which included extended sequencing, strongly suggested that all cases derived from a single importation of measles virus genotype D4. The use of timely genotype sequencing, rigorous epidemiologic investigation, and a better understanding of the gaps in surveillance are needed to maintain Ontario’s measles elimination status.

In Canada, the last endemic measles case was reported in 1997, and elimination status was achieved the following year (*1*,*2*). This status is maintained as long as measles virus does not establish a chain of transmission spanning >12 months within a region (*3*). Against the background of elimination, a detailed travel history from measles case-patients is crucial to determine the probable source of infection. Laboratory investigation, including virus identification and genotyping, is also critical. Molecular epidemiologic analysis can provide information about transmission patterns of circulating virus strains and help identify potential sources of infection (*3*).

The high risk for measles importation because of diverse and globally connected communities and the high infectivity of the measles virus make maintenance of immunity within the Ontario population critical. Two doses of measles vaccine is the most effective method of preventing disease, and elimination can only be achieved and maintained with high vaccination coverage. Two-dose measles-containing vaccination coverage in Ontario was estimated at 88.3% among 7-year-olds and 95.4% among 17-year-olds during the 2012–13 school year (*4*). Two-dose vaccination coverage of >95% is recommended to achieve measles herd immunity (*5*,*6*).

We describe an unusual measles outbreak that occurred in Ontario, Canada, in 2015. Our analysis focuses on the outbreak response and laboratory findings.

## Methods

### Epidemiologic Investigation

Measles is a reportable disease in Ontario, requiring physicians and laboratories to notify local Ontario public health units immediately of all suspected and confirmed cases. Data regarding cases are captured in the provincial reportable disease database, the integrated Public Health Information System (*7*). We analyzed data on all measles outbreak case-patients with rash onset dates during January 25–March 23, 2015. Confirmed cases were defined according to the provincial measles case definition (Technical Appendix) (*8*), and then according to an outbreak case definition, to exclude imported index cases and facilitate monitoring of potential outbreak cases. We also describe case-patient and contact investigation by public health unit, focusing on the 2 public health units handling the most cases, given that policies varied by health unit. Immunization information was acquired through interviews with case-patients or their legal guardians; information was validated through 1 of 2 provincial immunization repositories or providers. We obtained ethics approval from Public Health Ontario’s research ethics board.

### Laboratory Testing

Urine, throat swab, or nasopharyngeal swab specimens were collected from persons with a compatible clinical illness and submitted for molecular testing to Public Health Ontario Laboratories (PHOL), which performs frontline measles diagnostic testing. Total nucleic acid extraction was performed by using the NucliSens easyMAG extraction system (bioMérieux Canada Inc., Québec, Canada). One-step real-time reverse transcription PCR (rRT-PCR) was performed by using the ABI PRISM 7900HT Sequence Detection System (Applied Biosystems, Foster City, CA, USA) and the TaqMan RNA-to-Ct 1-Step Kit (Life Technologies Corporation, Carlsbad, CA, USA). A previously published rRT-PCR protocol was used (*9*). Detection of >1 of the gene targets is considered sufficient for laboratory detection of measles virus. Serum or plasma specimens submitted for diagnostic serology were tested for measles virus IgM and IgG by using an ELISA test kit according to the manufacturer’s instructions (Euroimmun, Luebeck, Germany).

Urine, throat swab, or nasopharyngeal swab specimens collected from laboratory-confirmed case-patients were referred to the National Microbiology Laboratory (NML) for genotyping. Total nucleic acid was extracted by using the QIAamp Viral RNA kit (QIAGEN, Valencia, CA, USA) or the MPLC Total Nucleic Acid Isolation Kit–High Performance on the MagNA Pure LC 2.0 (Roche Applied Science, Indianapolis, IN, USA). The World Health Organization (WHO) standardized genotyping regions (450 nt of the nucleoprotein gene [N-450] and 1,854 nt of the hemagglutinin [H] gene) were amplified (*10*) with primer pairs MVN1109/MVN1698R (Technical Appendix Table), H1/H6, and H5/H2 (modified from Kessler et al. [*11*] by using the QIAGEN OneStep RT-PCR kit. Purified amplicons were sequenced by using amplification primers and H gene internal primers H3, H4, H7, and H8 (also modified from Kessler et al. [*11*]). The hypervariable noncoding region between the matrix and fusion genes (MF-NCR) (i.e., 1,024 nt, from the stop codon of the matrix gene [nucleotide 4,443 of MVi/New York.USA/26.09/3; GenBank accession no. JN635402.1] to the start codon of the fusion gene [nucleotide 5,466 of MVi/New York.USA/26.09/3; GenBank accession no. JN635402.1] inclusive) was amplified in 1 fragment (primers 4200f/5609r) or in 2 overlapping fragments (primers 4200f/4869r and 4801f/5609r) (where additional sequencing primers also are noted; Technical Appendix). Raw sequence data were assembled and trimmed by using SeqMan Pro software (DNASTAR, Madison, WI, USA) and maximum-parsimony phylogenetic trees generated by using MEGA6 software (*12*). Genotypes were assigned by highest homology of N-450 sequences to WHO genotype reference sequences (*11*). Rapid genotyping for vaccine strains (genotype A) was performed for 30 clinical specimens from 25 patients by using a genotype A–specific rRT-PCR developed in-house (A. Severini, pers. comm.) and confirmed by standard genotyping.

### Statistical Analyses

We calculated incidence rates by using 2015 demographic data from Statistics Canada obtained through IntelliHealth Ontario. Descriptive analyses were performed by using Microsoft Excel 2010 (Microsoft, Redmond, WA, USA). Where appropriate, we excluded cases with missing data from analyses.

## Results

### Descriptive Epidemiology

#### Overall

Nineteen measles cases were reported during the period under surveillance; however, 1 case was excluded because it did not meet the outbreak case definition (an imported case in a patient with a travel history to Pakistan and a rash onset occurring after the outbreak was underway). Therefore, 18 confirmed cases of measles with rash onset occurring January 25–February 17, 2015, were reported by 4 public health units and met the outbreak case definition (Figure), representing an overall incidence of 1.3 cases/1 million population. Case demographic and immunization data were summarized (Table 1); 61% of case-patients were adults. Immunization status was known for 14 of the 18 case-patients, and of these, most (64.3%) were unimmunized (3 adults and 6 children). The 2 fully immunized case-patients were adults (Table 2). Two adult case-patients were hospitalized, and a third sought emergency department treatment. All case-patients recovered without complications.

**Figure:**
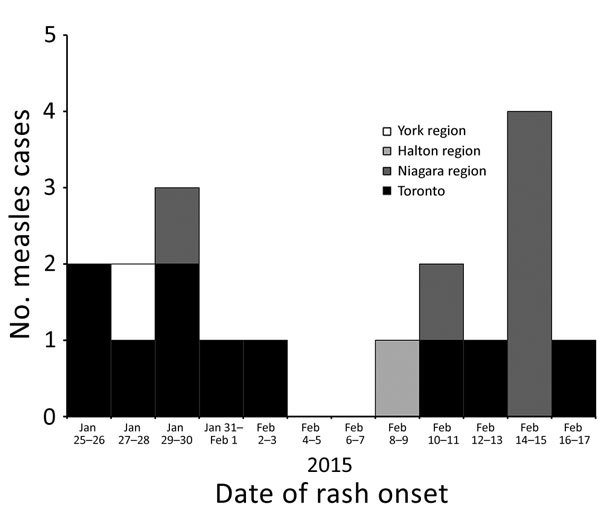
Number of measles outbreak cases, by date of rash onset, Ontario, Canada, January 25–March 23, 2015.

**Table 1:** Demographic characteristics of 18 case-patients identified during a measles outbreak, by health unit and overall, Ontario, Canada, January 25–March 23, 2015*

Characteristic	No. (%) patients			
	NRPH	TPH	Other PHU	Overall
Age group, y				
<18	4 (66.7)	3 (30.0)	0	7 (38.9)
>18	2 (33.3)	7 (70.0)	2 (100)	11 (61.1)
Sex				
M	2 (33.3)	4 (40.0)	2 (100)	8 (44.4)
F	4 (66.7)	6 (60.0)	0	10 (55.6)
Hospitalized				
Yes	1 (16.7)	1 (10.0)	0	2 (11.1)
No	5 (83.3)	9 (90.0)	2 (100)	16 (88.9)
Immunization status				
Unknown	0	3 (30.0)	1 (50)	4 (22.2)
Known	6 (100.0)	7 (70.0)	1 (50)	14 (77.8)
Unimmunized	6	3	0	9
1 dose	0	3	0	3
2 doses	0	1	1	2

*NRPH, Niagara Region Public Health; PHU, public health unit; TPH, Toronto Public Health.

**Table 2:** Selected characteristics of 18 case-patients identified during a measles outbreak, Ontario, Canada, January 25–March 23, 2015*

Case-patient no.	Date of rash onset	Age, y	Health unit	Immunization status, no. doses	IgM	Sequencing of N-450, H gene, and MF-NCR†	
1	Jan 25	22	Toronto	0	+	Type sequence to which sequences from all other cases are compared	
2	Jan 26	39	Toronto	1	+	1 SNP in the H gene (C961T)	
3	Jan 27	1	Toronto	0	IND	No changes	
4	Jan 27	24	York	2	+	No changes	
5	Jan 29	20	Niagara	0	+	No changes	
6	Jan 29	55	Toronto	Unknown	+	H gene sequence not determined; otherwise no changes	
7	Jan 30	1	Toronto	0	NT	1 SNP in the H gene (C1497T)	
8	Jan 31	39	Toronto	2	+	1 SNP in the MF-NCR (G932A)	
9	Feb 2	46	Toronto	Unknown	+	No changes	
10	Feb 9	35	Halton	Unknown	+	No changes	
11	Feb 10	14	Niagara	1 (PEP)	–	No changes	
12	Feb 11	34	Toronto	1	+	No changes	
13	Feb 13	2	Toronto	Unknown	+	No changes	
14	Feb 14	10	Niagara	1 (PEP)	NT	No changes	
15	Feb 14	17	Niagara	1 (PEP)	NT	No changes	
16	Feb 14	10	Niagara	1 (PEP)	NT	No changes	
17	Feb 14	23	Niagara	0	NT	Not determined (specimens unavailable)	
18	Feb 17	41	Toronto	1	+	No changes	

*H, hemagglutinin; IND, indeterminate; MF-NCR, noncoding region between the matrix and fusion genes; N-450, 450 nt of the nucleoprotein gene; NT, not tested; PEP, postexposure prophylaxis, SNP, single-nucleotide polymorphism. †All sequence results are in reference to the earliest detected sequence: MVs/Ontario.CAN/3.15 (GenBank accession nos. KU218405, KU218406, and KX396596 for the N-450, H gene, and MF-NCR, respectively), which is designated the “type sequence” for the purposes of this outbreak. The N-450 sequences, which were identical for all 17 cases with sequences, did not match any named lineage in MeaNS, the World Health Organization’s measles sequence database.

#### Toronto Public Health

On January 28, 2015, the first measles case of this outbreak was reported to Toronto Public Health (TPH). Three additional cases were reported during the following 3 days, triggering extensive public health investigations, a local outbreak declaration, and activation of the Incident Management System within TPH to help manage the response. Six additional cases were confirmed during the following 3 weeks, yielding a total of 10 cases (Figure). 

Each case-patient was asked about daily activities during the 21 days before symptom onset (*13*) to identify locations where the virus might have been acquired or transmitted. Initial case-patient interviews focused on travel, contact with others who traveled or anyone known to be ill with measles, and visits to healthcare providers or hospitals. A list of acquisition and transmission exposures was updated daily and used to inform questions during interviews of subsequent case-patients and to search for epidemiologic links between cases.

None of the confirmed case-patients reported travel to a measles-endemic area. Some community exposures were identified for subsets of cases, but evidence was insufficient to confirm links in both place and time. Because epidemiologic links were not identified through traditional methods, 3 case-patients were reinterviewed with an exposure-focused questionnaire. The questionnaire was supplemented by reference to publicly available social media information (e.g., Instagram posts) to facilitate recall and by requests to review online banking records to identify additional acquisition and exposure locations. Concurrently, a daily updated list of potential acquisition and transmission exposures from the previous day’s interviews was imported into Pajek social networking software (*14*) to automate the identification of overlapping exposures. This step increased efficiency and ensured a more systematic search for potential linkages. Nonetheless, no epidemiologic links could be confirmed for TPH’s cases. Given the rash onset dates (January 25–February 17, 2015), there might have been 2 or 3 generations of cases (*15*).

TPH investigated an additional 140 suspected measles cases during the outbreak period. Clinical presentations of patients with suspected infection were compared with the provincial case definition, and clinical samples were collected from these patients to confirm or rule out measles. Patients were provided with health education and asked to temporarily self-isolate from group settings. In a break from usual policy, persons living in Toronto who were recently vaccinated before onset of a measles-like rash and suspected of having an adverse event after immunization were investigated by genotyping to rule out the possibility of an actual measles infection, given that measles might be circulating in parts of the community.

TPH identified 1,548 contacts who were potentially exposed to the 10 confirmed measles case-patients, including 223 persons who resided outside of Toronto. Almost all (96.7%) were associated with exposure at healthcare institutions, with the number of contacts identified ranging from 3 at a physician’s office to 414 at a major acute care hospital. In addition, 51 contacts (3.3%) were identified in association with noninstitutional exposures. Contacts were categorized as high- or low-risk, according to Ontario Ministry of Health and Long-Term Care protocol (*7*) and TPH Measles Policy. High-risk contacts included household contacts, susceptible pregnant women, infants <12 months of age, immunocompromised persons, healthcare workers, or children 1–6 years of age who might not have received their second dose of measles, mumps, and rubella (MMR) vaccine. All other contacts were considered low-risk. High-risk and household contacts were interviewed by telephone or a home visit. Low-risk contacts were notified of their exposure and provided with information and directions in letters couriered to their home addresses. A measles hotline was established to take calls from healthcare providers, contacts who had received letter notification, or the general public. The line received 280 calls during its period of operation.

In addition to making 578 phone calls and sending 808 exposure notification letters, TPH issued 153 exclusions to unimmunized and underimmunized persons associated with high-risk settings (e.g., daycare attendees and healthcare workers) and provided either MMR vaccine or immunoglobulin as postexposure prophylaxis for 132 persons, depending on the timing of the exposure and risk condition in the contact, according to provincial guidelines (*7*). No known secondary cases were found among identified contacts.

#### Niagara Region Public Health

During the outbreak period, Niagara Region Public Health (NRPH) identified 6 confirmed cases of measles (Figure) and investigated links among the cases. Five cases had laboratory confirmation and the remaining case had an epidemiologic link to a laboratory-confirmed case. The index case-patient had traveled to Toronto during her incubation period. Although no direct link could be found to any TPH case-patient, the NRPH patient traveled on public transport and went to a large entertainment venue while in Toronto. The other 5 case-patients, all of whom were unimmunized, were family members of the index case-patient and were deemed secondary cases. Four of the 5 secondary case-patients received 1 dose of MMR vaccine after exposure and before becoming case-patients; however, these doses were not administered within 72 hours. An additional 25 suspected cases were reported and investigated by NRPH during this period; none met the case definition.

NRPH staff identified and followed up on a total of 1,837 contacts who were potentially exposed to the 6 case-patients. Two exposure sites outside of the region were identified, and additional notification was given to the other health units involved. Most (88%) of the Niagara contacts were associated with exposure at schools and school-related activities. Contact management was conducted by telephone, record review, or in-person interview. Panorama, the provincial immunization repository, was used to identify that 79 NRPH students were either not fully immunized or did not have complete immunization records reported. Health education and recommendations were provided to all students. Most students (55 [70%]) submitted appropriate documentation, whereas 24 (30%) received exclusion letters and were not able to return to school until NRPH received documentation that the student was fully immunized. A total of 279 contacts (15%) who were associated with other exposure settings (e.g., private residences, community centers, retail stores, and healthcare institutions) were identified. Contact management involved a review of the contact’s measles susceptibility, immunization status, and health education and recommendations for further action (e.g., vaccine), if needed.

In February 2015, a total of 367 doses of MMR or measles, mumps, rubella, and varicella (MMRV) vaccine were administered at NRPH general immunization clinics. This number is 350 doses more than the annual historical average during the previous 2 years. The increased demand resulted in increases in the capacity and service hours of the general immunization clinics and the addition of 3 supplementary MMR/MMRV vaccine–only clinics and 2 school-based clinics. Additionally, 4,826 doses of measles-containing vaccine were distributed to community health providers. This number is 3,885 more than the annual historical average during the previous 2 years. At the same time, staff received ≈12,000 incoming calls regarding measles, compared with the typical average of ≈3,700 calls per month. NRPH also made ≈8,000 outgoing calls for measles follow-up actions compared with the average of ≈3,200 calls per month. A measles hotline was activated to assist in managing call volumes.

#### Additional Cases

Two additional outbreak cases were reported among residents of Halton Region and York Region public health units (Figure). Both case-patients were male adults, and neither had recently traveled. One case-patient had received 2 doses of MMR vaccine, and the other had an unknown immunization status. Staff from these public health units instituted disease control measures to manage susceptible contacts and exposure settings and to rule out epidemiologic links to cases from TPH or NRPH.

### Laboratory Testing

#### Diagnostic Testing

During the outbreak period, PHOL received 966 specimens from 610 patients for measles rRT-PCR testing, including the 17 outbreak-related case-patients. Measles RNA was detected in 58 specimens from 36 persons; NML detected measles virus by PCR in specimens from 33 of those persons. Of the specimens in which measles RNA was detected by PCR at PHOL but not reproduced at NML, 1 was considered a false positive at PHOL, and 2 had received measles-containing vaccine before onset of symptoms and, like most patients with vaccine-associated cases, probably had viral loads near the threshold for detection and therefore were missed upon retesting at NML. NML identified wild-type virus in 17 of 33 persons and genotype A vaccine strain by conventional N-450 sequencing in the remaining 16. In addition, NML rapidly detected vaccine strain in 15 of these 16 persons by using a laboratory-developed rRT-PCR specific for measles vaccine strain, providing genotypic information up to several days before traditional N-450 sequencing.

A total of 1,484 serologic specimens were submitted for diagnostic testing (IgM and IgG) during the outbreak period, compared with 262 specimens during the corresponding period in 2014. In addition, 34,708 specimens were submitted to check immune status (IgG serologic tests only), representing a 155% increase in submissions to PHOL compared with the same period in 2014, when 13,606 specimens were received for immunity screening. In total, 47 (3%) of the 1,484 specimens submitted for IgM testing were reactive. IgM serologic specimens were submitted for 14 of the 17 PCR-confirmed measles cases; 11 of these 14 cases were IgM reactive, and 1 was IgM indeterminate. Among the 8 PCR-positive patients with measles vaccine strain, 6 were IgM positive, 1 was IgM indeterminate, and 1 was IgM negative. Excluding the 7 IgM-reactive specimens in persons documented to have been shedding vaccine strain, most (28/40 [70%]) of IgM-reactive specimens reported did not represent measles infection.

#### Genotyping

Seventeen outbreak-related measles cases were genotyped using the WHO-recommended targets to gather evidence of relationships between cases and the possible outbreak origin. All cases were genotype D4 infections, with identical N-450 sequences (GenBank accession no. KU218405). Full-length H gene sequences (1,854 nt) were successfully obtained from 16 case-patients. Sequences from 14 case-patients were identical to each other (GenBank accession no. KU218406), whereas 2 were each 1 nucleotide different from the majority sequence (Table 2). A search of the N-450 sequences deposited into MeaNS, the WHO measles sequence database (*16*), revealed that the identified D4 viral strain was not associated with any reported measles activity globally, although it had been identified in 2 contemporary imported cases in New York state (GenBank accession nos. KP797976 and KP797977). In the absence of clear travel history or an epidemiologic link between most case-patients, extended genotyping of the MF-NCR was performed to seek additional evidence about possible chains of transmission. With the exception of 1 sequence that differed by a single nucleotide (Table 2), all cases had identical MF-NCR sequences (GenBank accession no. KX396596) and shared a characteristic previously unreported pattern of insertions and deletions.

## Discussion

This outbreak was unusual as no travel history or common case-patient exposures were noted, despite intensive investigation, and laboratory evidence strongly suggested that all cases formed the same chain of transmission. Despite rapid increases in reported cases early in the outbreak, the total number of cases was relatively small and transmission was limited. The relatively high immunization coverage in Ontario (*4*) probably played a role in this respect; most cases occurred in either unimmunized people or those who were not fully immunized. This outbreak occurred in an area of the province where there is a large multicultural population and a high volume of international travelers (*17*). Limited transmission might also reflect that the outbreak occurred in areas of Ontario with a high proportion of immigrants from measles-endemic countries, who are more likely to have natural immunity from previous infection (*17*).

We never identified a source case in this outbreak, and an interval of >5 months elapsed between report of the first case in the outbreak and report of the previous measles case in the province. Typically, we are able to identify linkages with travel or potential exposure sites for recent measles cases in Ontario. Although some overlapping exposures were identified, no epidemiologic links could be confirmed among the index case in NRPH or any of the cases in Toronto, York Region, or Halton Region. This finding might reflect an exposure that involved a casual interaction not deemed worth mentioning by the case-patient but one that was actually critical given the highly communicable nature of the measles virus. Alternatively, a source case-patient might have been exposed other case-patients in several locations while moving through the city during the infectious period (i.e., a point-source case but not a point-source exposure). The absence of a source case indicates that not all cases were reported to public health (*15*,*18*)

Many clinicians might not consider a diagnosis of measles in a patient with measles-like symptoms, either because of lack of familiarity or because its rarity results in omission from the differential diagnosis. Clinicians might also be unfamiliar with appropriate diagnostic testing. During this outbreak, we produced guidance for physicians about best practices for assessing suspected measles cases (*19*). These guidelines indicated that most patients should receive measles-containing vaccine when doubt exists about their immune/immunization status, rather than serologic testing, given that serologic testing can delay protecting a nonimmune person and can result in unnecessary healthcare utilization (*19*). Despite this guidance, a large volume of samples were sent to PHOL for IgG serologic testing. IgM serologic testing is also problematic because there is a high likelihood of false-positive tests in a low-prevalence setting (*20*). Our findings substantiated this; >70% of the positive measles IgM tests were from persons who were not ultimately reported as having measles. Serologic testing cannot differentiate between the immune response after wild-type infection and recent immunization; therefore, it is not recommended for determining immunity in well persons during an outbreak or among persons who have been recently vaccinated.

All 17 cases that were genotyped using the WHO-recommended N-450 and H gene targets showed identical or minimally variable sequence. Because measles viral strains associated with large outbreaks globally can result in repeat importations of the same sequence, our findings cannot be taken as absolute proof of a single importation. A single nucleotide difference can be enough to suggest separate importation events (*21*). Consequently, the possibility of multiple importations cannot be excluded. Nonetheless, the hypothesis of a single importation was supported by sequencing of MF-NCR, which has been identified as a hypervariable region within the measles genome (A. Severini, pers. comm.). With the exception of 1 case that differed by a single nucleotide, this region was identical in this outbreak and showed a characteristic pattern of insertion/deletion. This pattern clearly distinguished this outbreak from those associated with the most closely related reported isolates MVi/New York.USA/26.09/3 and MVi/Florida.USA/19.09, strongly suggesting that all cases derived from a single importation event. The genetic diversity of measles virus decreases as progress is made toward global elimination. As a result, extended genotyping beyond the WHO standard targets, as was required in this outbreak, will probably be needed more often to define the molecular epidemiology of measles outbreaks (*22*).

In conclusion, until measles is eradicated worldwide, Ontario’s public health system continues to respond to measles activity. The level of response is challenging from a public health perspective. The use of timely genotype sequencing, rigorous epidemiologic investigation, and a better understanding of the gaps in surveillance are needed to maintain Ontario’s measles elimination status. Molecular epidemiologic analysis beyond WHO-recommended targets will probably play an increasing role in the future.

Technical Appendix Case definitions and primers used for the amplification and sequencing of the noncoding region between the matrix and fusion genes identified through molecular epidemiologic analysis during a measles outbreak, Ontario, Canada, January 25–March 23, 2015.
