# Supplementary material for: Measles Outbreak with Unique Virus Genotyping, Ontario, Canada, 2015
Source: Emerg Infect Dis. 2017 Jul;23(7):1063–9. doi: 10.3201/eid2307.161145 (PMC5512469; doi:10.3201/eid2307.161145)
Supplement: Technical Appendix — Case definitions and primers used for the amplification and sequencing of the noncoding region between the matrix and fusion genes identified through molecular epidemiologic analysis during a measles outbreak, Ontario, Canada, January 25–March 23, 2015. [file 16-1145-Techapp-s1.pdf]

Article DOI: https://dx.doi.org/10.3201/eid2307.161145

Measles Outbreak with Unique Virus Genotyping, Ontario, Canada, 2015

Technical Appendix

Case Definitions

Ontario measles case definition: cases with laboratory confirmation of infection with clinically compatible signs and symptoms in the absence of recent immunization with measles-containing vaccine, or clinically compatible signs and symptoms in cases that were epidemiologically linked to a laboratory-confirmed case (*1*). Laboratory confirmation consists of any of the following: positive measles virus culture; positive direct nucleic acid amplification test for wild type measles virus RNA; seroconversion or a significant rise in measles Immunoglobulin G (IgG) titer between acute and convalescent sera; or positive for measles Immunoglobulin M (IgM) antibody and an epidemiologic link to a laboratory confirmed case or a positive travel history. A probable case of measles is a case with clinically compatible signs and symptoms in the absence of recent immunization with measles-containing vaccine and positive (IgM) serology testing, or clinically compatible signs and symptoms in a person who has traveled to an area of known measles activity (*1*).

Outbreak Case Definition (Implemented February 20, 2015)

Confirmed or probable cases with rash onset dates between January 25, 2015 and March 23, 2015, residing in Toronto or Niagara region, or who were epidemiologically linked to confirmed measles cases in these health units and had no other known source.

Reference

1. Ontario Ministry of Health and Long-Term Care Infectious Diseases. Ontario Ministry of Health and Long-Term Care Infectious Diseases protocol appendix B [cited 2015 Dec 7]. http://www.health.gov.on.ca/en/pro/programs/publichealth/oph_standards/docs/measles_cd.pdf

Technical Appendix Table. Primers used for the amplification and sequencing of the MF-NCR region

Primer	Primer Sequence 5′–3′	Amplicon size
MeV4200f	GGC ACC AGT CTT CAC ATT AGA AG	1,409
MeV5609r	CGA GTC ATA ACT TTG TAG CCT GC	
MeV4200f	GGC ACC AGT CTT CAC ATT AGA AG	669
MeV4869r	CTT GGC CCT RAG TTT TGT TTA G	
MeV4801f	CAC AAG CGA CCG AGG TGA C	808
MeV5609r	CGA GTC ATA ACT TTG TAG CCT GC	
MeV4409f*	CAT AAA TGA TGA CCA AGG AC	
MeV4428r*	GTC CTT GGT CAT CAT TTA TG	
MeV4826r*	GTT GGG TCA CCT CGG TCG	
MeV5126f*	CAC ACA CSA CCA CGG CAA CC	
MeV5145r*	GGT TGC CGT GGT SGT GTG TG	
MVN 1109	GCT ATG CCA TGG GAG TAG GAG TGG	590
MVN 1698R	GGC CTC TCG CAC CTA GTC TAG	

*Sequencing primers
